# COVID-19 healthcare success or failure? Crisis management explained by dynamic capabilities

**DOI:** 10.1186/s12913-024-11201-x

**Published:** 2024-06-21

**Authors:** Ritva Rosenbäck, Kristina M. Eriksson

**Affiliations:** Department of Engineering Science, University West, Gustava Melins gata 2, Trollhättan, 46132 Sweden

**Keywords:** Dynamic capabilities, Healthcare, COVID-19 pandemic, Static capabilities, Crisis management

## Abstract

**Introduction:**

This paper presents a structured review of the use of crisis management, specifically examining the frameworks of surge capacity, resilience, and dynamic capabilities in healthcare organizations. Thereafter, a novel deductive method based on the framework of dynamic capabilities is developed and applied to investigate crisis management in two hospital cases during the COVID-19 pandemic.

**Background:**

The COVID-19 pandemic distinguishes itself from many other disasters due to its global spread, uncertainty, and prolonged duration. While crisis management in healthcare has often been explained using the surge capacity framework, the need for adaptability in an unfamiliar setting and different information flow makes the dynamic capabilities framework more useful.

**Methods:**

The dynamic capabilities framework’s microfoundations as categories is utilized in this paper for a deductive analysis of crisis management during the COVID-19 pandemic in a multiple case study involving two Swedish public hospitals. A novel method, incorporating both dynamic and static capabilities across multiple organizational levels, is developed and explored.

**Results:**

The case study results reveal the utilization of all dynamic capabilities with an increased emphasis at lower organizational levels and a higher prevalence of static capabilities at the regional level. In Case A, lower-level managers perceived the hospital manager as brave, supporting sensing, seizing, and transformation at the department level. However, due to information gaps, sensing did not reach regional crisis management, reducing their power. In Case B, with contingency plans not initiated, the hospital faced a lack of management and formed a department manager group for patient care. Seizing was robust at the department level, but regional levels struggled with decisions on crisis versus normal management. The novel method effectively visualizes differences between organizational levels and cases, shedding light on the extent of cooperation or lack thereof within the organization.

**Conclusion:**

The researchers conclude that crisis management in a pandemic, benefits from distributed management, attributed to higher dynamic capabilities at lower organizational levels. A pandemic contingency plan should differ from a plan for accidents, supporting the development of routines for the new situation and continuous improvement. The Dynamic Capabilities framework proved successful for exploration in this context.

## Introduction

The COVID-19 pandemic is a disaster [1]. However, it differs from many other disasters by the worldwide spread, the uncertainty about the patient treatment, especially in the beginning, and the long duration. The healthcare crisis management challenges in a long duration pandemic are different from management in short duration disaster like an earthquake or a major accident. The management in shorter crises or disasters is described in the research of surge capacity (SuC) [2, 3], but the COVID-19 pandemic revealed that successful management in a pandemic, needs to be different [4]. Further, pandemics differ from other long-duration disasters like war or severe air pollution, due to the uncertainty of the type of healthcare and knowledge needed. Merely, the infected patients appear at the hospital, thus the first to receive information about both the number of patients and their needs are the professionals at the hospitals [4]. Usually the flow of information comes from a rescue leader through the regional management that prioritizes and distributes the patients to the hospitals [5]. The hospital management needs to use the in-house knowledge and improve the mobility at the hospital [6]. Thus, the management’s need in a pandemic is less hierarchical and more learning and innovative [4, 7, 8].

SuC expresses the demand of unusually high capacity caused by crisis and disasters [2, 3]. The concept of SuC seems to be the base for the worldwide used NATO standard for crisis management, with a hierarchic structure and strong rules of communication [5]. Resilience (R) is the most used management framework in healthcare organizations, defined as the capacity to absorb shocks while maintaining function, focusing on two categories i.e., robustness and rapidity [7, 9]. The strategic “inside-out” Resource-based view, focus on how the resources on hand could be used to the market “inside-out” and have developed during time to the organization’s ability to renew competences to adjust to changes in the surroundings, and include understanding of the requirements from the market or environment (“outside-in”) [10]. The different flow of information and the constant need for learning and development in an unknown and continuously changing environment could make the hierarchic system of SuC too static and less successful. Therefore, in a disaster such as the COVID-19 pandemic other approaches to crisis management need to be considered. The Dynamic Capability (DC) framework was designed to explain how organizations achieve and sustain competitive advantages by adjusting resources and adapting to changing environments. Originating from a resource-based view, Dynamic Capabilities (DCs) emphasize an organization’s ability to adapt resources to new conditions. From this perspective the DC framework has been limited applied in healthcare management research before the COVID-19 pandemic [11–13]. However, the possibilities of DCs in the context of the public sector have gained research interest, e.g., Furnival et al. [12] suggest further research into using the microfoundations and Pablo et al. [13] ask for more research on how managers or organizations can enable DC in the public sector. The application of the DC framework in health care organizations are thus gaining research interest and to understand the applicability of DCs in health care, especially in relation to unpredictable and long duration disasters, further research into the field is called for. Contributions to the field, demonstrating results from in-dept studies with hospital management expertise at different management levels may be especially valuable for building knowledge toward meeting future long-duration disasters and crises with similar characteristics. This study adopts and develops the DC framework to investigate effective resource utilization and how the DC framework could be more usable, especially in long-duration pandemics. This prompts the research question: *How can the DC framework explain the disaster management in healthcare organizations during the COVID-19 pandemic?* The research presented develops the concept of the DC framework, which is applied to a multiple qualitative case study to understand the management changes during the COVID-19 pandemic.

The paper starts with an overview of applied crisis management theories, thereafter the results from a structured review of the use of SuC, R and DC in healthcare research, especially focusing of disasters and pandemics, is presented. The methodology of a qualitative multiple case study and the two cases are outlined and thereafter the findings are reported. The discussion and conclusion wrap up the paper.

## Crisis management in literature

SuC expresses the demand of unusually high capacity caused by crisis and disasters [2, 3]. SuC have been studied over the last decade, mostly in healthcare organizations, but can be generalized to other systems involving complex activities [9]. The management part of SuC is carefully stated with solid rules concerning how and to whom to communicate and incorporates a hierarchy of decisions [5].

R was originally used to describe ecological systems’ ability to resist disturbances [9]. The theories of R have been developed in crisis management science with the aim of improving performance of systems during crisis. R should include all resources that need to be safeguarded from expected or unexpected disturbances and can be described both as being robust during change, but also as the ability to absorb uncertainty [9]. Kruk, et al. [14] describe the need during the outbreak of a disease or other disasters resulting in a surge demand for healthcare. The conclusion is that a resilient health system needs to be aware, diverse, self-regulating, integrated, and adaptive [14]. During the COVID-19 pandemic, R could be described by three required preconditions; global solidarity, legal framework, and workforce policies [15], which are aligned with the research of Kruk et al. [14] and Therrien et al. [9]. McDaniels et al. [7] recommend using R instead of SuC in healthcare organizations, due to the described less static management.

DCs focuses both on the perspective of how the market (outside) influences the organization (inside) and the perspective that the organization needs to adapt to the chosen market [16], but also to the inside-out perspective which values the organization’s knowledge and resources in the choice of strategy and marketplace [10, 17]. Teece, et al. [18], considered founders of the DC framework, describe the resource based view as static, when organizations in the short term are stuck with existing knowledge and structure. DCs are a special class of capabilities that describe change and innovation essential when organizations need to sustain performance in a changing environment [19]. The aspect of cyclicity and moving through the DC phases in several iterations may be necessary for organizations to be able to continuously develop [12] and reach a higher level of understanding of their specific organizations planning characteristics, such as shown by Eriksson, et al. [20]. Pablo et al. [13] describe this iteration to learn and transform as experimenting.

Further, the importance of taking a holistic view of the organization is stressed as a prerequisite when moving towards the capability of transformation [20]. Developed DCs are difficult for competitors to replicate and will give a competitive advantage and innovative response in a rapidly changing market when time to market is critical [18]. Both inside-out and outside-in strategy capabilities need to be dynamic and constantly renewed [21]. For moderately dynamic markets it is possible with traditional routines to build on predictable and analytical processes and build DC from existing knowledge. However, for high-velocity markets, with unpredictable outcomes, DCs need to develop to be simpler, more experimental, and iteratively relying on situation specific knowledge within simple rules and are often described vaguely as *“routines to learn routines”* [11]. Capabilities that are not supporting changes is by a few scholars called static capabilities (SC), e.g., Dawson [22] is using SC for exploring knowledge management and Mortensen et al. [23] are using it to explore barriers for futures literacy. The DCs have advanced in different areas and hereafter the development over the last ten years in healthcare disaster management are focused and described.

## Crisis management in healthcare literature

**Fig. 1:**
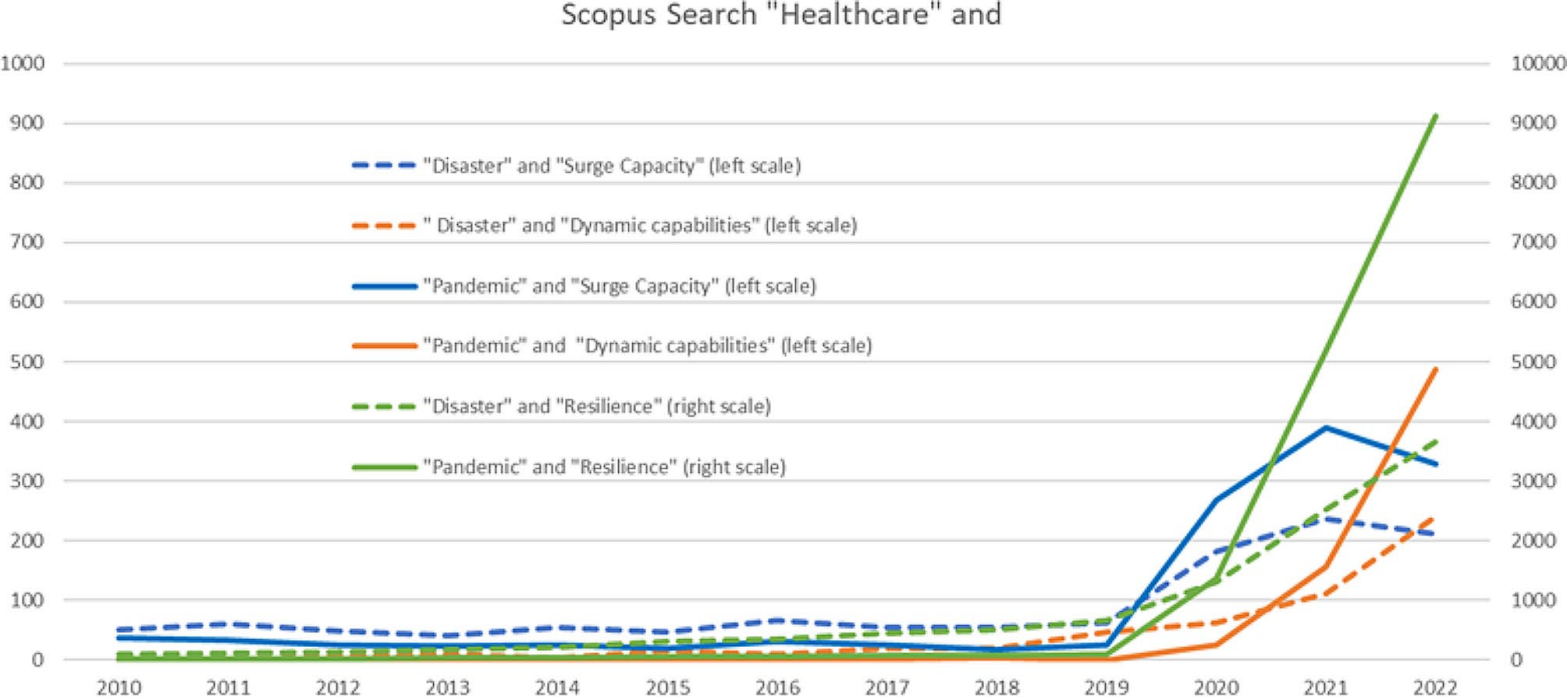
Use of the crisis management concepts SuC, R and DCs

The COVID-19 made the healthcare business volatile and has caused an exponential increase in frequency of use of concepts of crisis management i.e., SuC, R, and DCs. A structured search in Scopus, searching “all fields” with the keywords “Healthcare” and “Disaster” (doted lines) or “Pandemic” (full lines) and “Surge Capacity”, “Resilience” or “Dynamic Capability” between 2010 and 2022, delivers a result of the amount of research papers applying the concepts, see Fig. 1. Research studies investigating the use of R is more than ten times higher than SuC and DCs (left scale) and shall therefore be read at the right scale in Fig. 1. SuC and R seem to have been used in healthcare crisis management research at least from the beginning of 2010th decade both for pandemics and disasters. The interest of R seems to rise in use especially in combination with disasters and the interest of DC started later, but the use in research increased after 2014. At the start of the COVID-19 pandemic in 2020 the research into all three concepts increased largely and DCs is the concept with the highest increase in publications between 2019 and 2022 (> 400 times) after which it exceeded the use of SuC. SuC declined between 2021 and 2022. Thus, exploring the DC concept in healthcare was found interesting.

The search in Scopus was limited from all fields to; article title, abstract and the keywords was reduced to “healthcare” and “Dynamic capabilities” resulting in 88 papers (reduced from 5134 results). Further papers in the areas of computer science, focusing on simulations and analytics, were omitted, resulting in 54 papers. The abstracts of those 54 papers were read and 24 papers of the highest relevance were kept. All 24 papers were read in full, and the eight most interesting papers were studied in more detail in this research. In addition, five research papers, found outside of the Scopus search through snowball technique, were included because of additional interesting and highly relevant research. Thus, in total 13 papers, outlined in Table 1, about DC in healthcare crisis management were studied in detail and used in the research presented in this paper.

**Table 1:** Papers found about DC in healthcare crisis management and used in this paper

	Scholars	Title	Journal
From Scopus	Evans, J. M., Brown, A. and Baker, G. R [19].	Organizational knowledge and capabilities in healthcare: Deconstructing and integrating diverse perspectives	*SAGE open medicine*, 2017.
	Furnival, J., Boaden, R. and Walshe, K [12].	A dynamic capabilities view of improvement capability”	*Journal of Health Organization and Management*, 2019.
	Linden, A. I., Bitencourt, C. and Muller Neto, H. F [31].	Contribution of knowing in practice to dynamic capabilities	*The Learning Organization*, 2019.
	Ljungquist, U [29].	Unbalanced dynamic capabilities as obstacles of organisational efficiency: Implementation issues in innovative technology adoption	*Innovation*, 2014.
	Loureiro, R., Ferreira, J. J. and Simoes, J [25].	Understanding healthcare sector organizations from a dynamic capabilities perspective	*European Journal of Innovation Management*, 2023.
	Ohrling, M., Solberg Carlsson, K. and Brommels, M [8].	No man is an island: management of the emergency response to the SARS-CoV-2 (COVID-19) outbreak in a large public decentralised service delivery organisation	*BMC Health Services Research*, 2022.
	Pablo, A. L., Reay, T., Dewald, J. R. and Casebeer, A. L [13].	Identifying, enabling and managing dynamic capabilities in the public sector	*Journal of management studies*, 2017.
	Sirmon, D. G., Hitt, M. A. and Ireland, R. D [31].	Managing firm resources in dynamic environments to create value: Looking inside the black box	*Academy of management review*, 2007.
Snowball	Sheng, M. L [27].	A dynamic capabilities-based framework or organizational sensemaking through combinative capabilities towards exploratory and exploitative product innovation in turbulent environments	*Industrial Marketing Management, 2017.*
	Karali, E., Angeli, F., Sidhu, J. S. and Volberda, H. [26[	Understanding healthcare innovation through a dynamic capabilities’ lens	*Healthcare entrepreneurship*, 2018.
	Sundararaman, T., Muraleedharan, V.R. and Ranjan, A [15].	Pandemic resilience and health systems preparedness: lessons from COVID-19 for the twenty-first century	*Journal of Social and Economical Development, 2021.*
	Chokshi, A. and Katz, H [30].	Emerging Lessons From COVID-19 Response in New York City	*The Journal of the American Medical Association, 2023*
	Boin, A., Hart, P., Stern, E., and Sundelius, B [32].	The Politics of Crisis Management: Public Leadership under Pressure	Cambridge University Press, Cambridge, 2016

### Dynamic capabilities framework in healthcare

The DCs framework is usually divided into sensing, seizing and transformation [24]. However, other scholars express it differently as i.e., detection, understanding and reconfiguration [25] or i.e., dynamic managerial capabilities and dynamic organizational capabilities, where the latter is divided as described above, but the former divides into managerial cognition, managerial human capital, and managerial social capital [26]. Moreover, Sheng [27] divides the capabilities into three groups for the inside-out view. First the “system capabilities” with the content of written regulations, guidelines, and instructions. Secondly the “socialization capabilities” can be explained as the organizations shared ideology and basic values and influences how the members of the organization treat each other in a crisis. The third is expressed as “coordinating capabilities” and influences the number of fruitful contacts in the organization. For the outside-in view Sheng [27] describes “organizational sensemaking”, as a continuous process of how the organization is seeking information of the environment and how this is formed to common goals for the organization. Moreover, in a framework for decision-making in crisis in major projects, sensing is explored as an important framework category [28] In the developed method in this paper Teece’s [24] the microfoundations are used as framework categories i.e., sensing, seizing and transformation.

Sensing includes the identification of all kinds of risks and opportunities, e.g., technical advancements, suppliers’ possibilities to deliver and regulations, preferably before they arrive [12, 29]. Research concerning sensing often refers to analytical and forecasting [30], and the need for real time data [8]. The capability of sensing focuses on service users, stakeholders, and suppliers [12] or on specific important factors e.g., problems detection, lack of coherence of safe routines or risk for high demand or exhaustion [31]. Ohrling et al. [8] describe the importance of rapidly understanding the unexpected during the COVID-19 pandemic and finding resources to increase the ability to analyze the situation and add that knowledge and experience to the emergency management team. Further, the communication to spread an always changing target and new information to the emergency management team and to everyone, to create a common understanding [8] could also be included in the DC of sensing. To make the sensing appear, meetings need to be highly frequent both in the organization and between organizations. However, it could be important to limit information due to a high and intense flow from different resources that may lead to misunderstandings [8].

Seizing can be seen as the enablers to make dynamic capabilities work and can both be already existent in the organization or newly developed. The DC of seizing provides a link between environmental change and internal adaptability [13] or it could be routines and processes for change [29]. A beforehand made contingency plan can be a part of the seizing; thus, these are often built on SuC and are therefore rather static and work against DC [32]. Seizing could also include culture and management capabilities in the managers’ choice of the competing priorities [12]. Routines could be how planning, evaluating and decision making should be done, how ideas are received and accredited and how leadership and teamwork are functioning in the organization [31]. Decentralization and a culture of rapidly responding from the information towards actions and more practically, routines and processes that enable higher frequency meetings, faster coordination, added experts and teamwork can be seen as parts of the DC of seizing [8].

The transformation includes implementing new processes and policies, for example, decentralization, co-specialization, or governance, and measuring improvement activities and reviewing plans and strategies [12, 29]. . Moreover, some researchers refer to learning to respond to changes [31]. The transformation during a pandemic needs to be continuous with adjustments and rearrangements, due to changing information and environment and the activities need to be tightly followed and continuously evaluated to build flexibility [8]. The sensing, seizing and transformation as described here is hereafter used in this research.

The synchronization of microfoundations is necessary to make the DC perform [33]. An organization without seizing will become cosmetic and bureaucratic, and therefore ineffective to take decisions and fulfill the customer needs due to shortage of inter-relationships between the microfoundations. Further, a shortage of transformation will ensure customers and stakeholders that the service will be provided, but it never happens. Without sensing, the organization will appear arrogant and unwilling to seek ideas and knowledge from the outside, thus just focusing on internal plans and strategies [12]. Whereas, a strong sensing capability could lead to high expectations of seizing and transformation, causing a capability gap, which could be recognized by a lack of top management [18, 29]. Moreover, they also mean that a strong sense and a strong transformation at local organizational level implies local unit-focused initiatives, thus, may suboptimize the local unit and not benefit the whole organization. If sensing and seizing capabilities are high, it leads to high barriers between local units, but could also lead to barriers between local units and the top management [29]. At the daily level, especially in healthcare, the transforming capability is strongest, and the staff will try their best to help the patients. However, a focus on operational tasks may lead to organizations with difficulties in verifying their capacity for change and responding quickly to changes in the surroundings [34]. Furnival et al. [12] suggest that organizations in a disaster are different, thus sensing will be more important to be able to rebuild organizational confidence and capability of movement. However, in non-crisis organizations, seizing may be of higher importance, where commitment and culture should help ensure continuous development.

### Methodology for the case study and case description

The methodology applied in the research presented is multiple case study. The case study methodology includes the collection of internal hospital documentation, documentation from externa public sources and qualitative data collection through interviews. The case studies are considered suitable when capturing different and elusive aspect and perspectives from real context [35, 36]. Thus, the method was chosen to capture and develop an encompassing view of capabilities for disaster management during the COVID-19 pandemic. The selection of case hospitals was meticulous. Several hospitals were considered before finalizing the choices [36, 37]. Key diversity factors included hospital size, infection pressure, pandemic timing, collaboration ability, and management stability. The first case, a medium-sized hospital, faced high and early infection pressure, could transfer patients, and had a stable organization. The second case was chosen for its contrasting attributes: larger size, lower and later infection pressure, responsibility to assist other hospitals, and a recent management change. To understand the selected cases, internal documents regarding mission and organization both before and during the Covid-19 pandemic were studied. Further, documents from external public sources were gathered and studied, e.g., newspaper articles and information from national press conferences during the pandemic to increase the knowledge about the pandemic situation and the cases.

The multiple and qualitative case study was built on semi-structured interviews with managers that were conducted about a year after the start of the COVID-19 pandemic. The choice of semi-structured interviews as data collection method were considered valuable for the multiple qualitative case study, to gain focused data and the managers personal view of the management [36]. Case studies produce context-dependent knowledge, and the data could be used to understand the complex issues of the aspects of the managers dynamic management during the pandemic [38]. The narratives from managers of different levels were used to identify their opinion of the organization’s management practice.

#### Case descriptions

The first investigated case (A) is a middle-sized hospital with about 1300 employees, located in a large Swedish region, with several hospitals. The case hospital is an emergency hospital, but without an infection department and with few intensive care unit (ICU) beds. The increase of the COVID-19 infection rate in the catchment area was rapid in the beginning of the pandemic and sometimes the percentage of hospitalized citizens was the highest in the country [39]. The hospital was about to implement a new NATO standard with instructions for starting a regional command center (RCC) at the regional headquarters and local command centers (LCC), with static rules for how to communicate and make decisions [5] and concluded the implementation during the beginning of the pandemic.

The second case hospital (B) was chosen to be different, as sought to be advantageous for designing a multiple case study [36]. Case hospital B is the central hospital in a less populated region (compared to Case hospital A). This region also includes two local hospitals. Case B has about 5000 employees and have an infection department and the most ICU beds in the region. Just before the COVID-19 pandemic the healthcare director was replaced and the region was reorganized and a regional organization was implemented with some of the department’s management centralized to the main hospital, for example the departments of infection and the departments of ICU. The contingency plan was not updated to the new organization.

#### Interviews

The interview sessions started in March 2021, one year after the onset of the pandemic, and were completed within a month for Case A and another month for Case B. At Case A, a total of twelve interviews from three organizational levels i.e., hospital manager group (3), department manager group ( 5) and unit manager group (4), were conducted. The presentation of the interviewees is found in Table 2 including the time of the interview. At case B, with a total of eight interviews were performed the hospital management were merged to a regional healthcare management group with the responsibility of the departments, directly reporting to the director of healthcare and hospital managers were not existing. Important functions were found at the regional level and therefore the three levels of management studied became i.e., regional manager group (RM, 3), regional healthcare manager group (2) and department manager group (3) and in total eight interviews were conducted. The presentation of the interviewees is found in Table 2 including the time of the interview.

**Table 2:** Interviewees in management groups and the length of interviews

Management group	Case A	Length [h: min]
Hospital managers (3)	Hospital manager (CEO)	1:15
	Chief physician 1	1:09
	Chief physician 2	0:55
Department managers (5)	Department manager of surgery	0:59
	Department manager of medicine and geriatrics	0:52
	Department manager of intensive care and surgery services	1:37
	Department manager of inpatient care	1:02
	Department manager ED	1:12
Unit managers (4)	2 Unit manager of ED	1:05
	Unit manager of inpatient geriatric	0:39
	Unit manager of inpatient care	0:55
	Unit manager of Surgery/ICU	1:07
	Case B	
Regional managers (3)	Chief of Staff of RCC	1:03
	Chief Hygiene Physician	1:17
	Chief Medical Officer with Preparedness Responsibility	1:10
Health care managers (2)	Regional healthcare director	0:55
	Chief of Staff of LCC	1:06
Department managers (3)	Department manager of internal medicine	0:56
	Department manager of infection	1:08
	Department manager of ED	1:21

The interviews were semi-structured, which means the interviewees were allowed to talk freely, and the interviewer avoided affecting the interviewees [37]. The same researcher moderated all interviews and used a semi structured interview guide as been described in earlier research [6], with topics of the feeling of the size of the disaster, the contingency plan, how they built capacity for the COVID-19 patients, management during the pandemic and the information flow, as support. Another researcher actively observed the interviews and used the interview guide to follow the completeness of the collection of information and sometimes added a few questions for completion. All interviews were conducted via video conferencing with both sound and video recording. The interviewees were later provided with feedback in the form of a lecture and a written report, to make sure the information gathered was correctly understood [40]. The recordings were verbatim transcribed and NVIVO14 was used to structure the data. Thereafter, the data were exported to Excel and further analyzed.

### Methodology analytical development

The DC and SC frameworks were applied and further developed in this study to explore and analyze the management during the pandemic in the cases. To align the data in relation to the DC and SC frameworks it was suitable to perform the analyses deductively. Therefore, the data were deductively analyzed by selecting excerpts, from the interviews, that aligned with the different DC framework categories (microfoundations)i.e., sensing, seizing and transformation, and SC framework categories (microfoundations), i.e., non-sensing, non-seizing, and non-transformation following other scholars’ definitions and proposals in their research. Moreover, to be able to receive deeper knowledge about the organization and the different management groups’ viewpoint of the organization’s performance at different organizational levels, the data was divided into organizational levels, i.e., department, hospital, regional and national level in case A. In case B one additional level of regional healthcare was used necessary by the special organization, where the regional healthcare organization worked besides the RCC stated in the contingency plan. The hospital level contained a spontaneously developed group of department managers during the first wave of the COVID-19 pandemic. However, during later waves a LCC was started as stated in the contingency plan and emerged with the department managergroup at the hospital level. Table 3 shows the 42 different categories in the deductive analyze.

**Table 3:** The framework categories coupled to organizational levels of the cases

Case	Organizational level	Framework category
A	Department level (DepLev) Hospital level (HospLev) Regional level (RegLev)	Sensing (Sens) Seizing (Seiz) Transformation (Trans) Non-Sensing (NonSens) Non-Seizing (NonSeiz) Non-Transformation (NonTrans)
B	Department level Hospital level Regional healthcare level (RegHealthLev) Regional level	Sensing Seizing Transformation Non-Sensing Non-Seizing Non-Transformation

The interviewee’s excerpts were analyzed several times both from the transcription of the interviews and later from the framework categories. This procedure was conducted to enhance the rigor of the research [41]. To be able to analyze and present the findings both qualitatively and quantitatively, the excerpts from each interviewee were only coded once to one framework categories.

The deductive analysis of the excerpts in the interviews to the framework categories of DCs and SCs at different organization levels was suitable and the researchers found the method satisfactory. The imposed quantitative analysis is done according to the visualizations in Table 4.

**Table 4:** Imposed quantitative analysis of deductively coded excerpts

Visualization	Shown Phenomenon	Figure in findings
Number of DC and SC excerpts/group of managers	The proportion of excerpts per management group to analyze the amount of bias in the data. The visualization also shows the opinion of the management groups if they found the total organization to be dynamic or static by the proportion of SC to DC.	2
Number of excerpts/organization level	The managers’ opinions about what organization level in the organization was the most dynamic or static. Also, here the proportion of SC to DC is showing the levels that was successful respectively failed in their disaster management.	3
Number of interviewees excerpts for each group of managers/organizational level	A more detailed picture of each management group’s opinion about the dynamic of each level of the organization. The results could visualize good cooperations, but also conflicts between the organizational levels.	4
Number and type of excerpts/organization level	The managers opinion about the number of each DS or SC is most at each organization level. Answers the questions; What level in the organization appropriate sensed the disaster? Who structured the work and used the competence and plans in a dynamic way? Who transformed most in the organization?	5, 6

### Findings: multiple case study

A qualitative analysis was conducted to clarify special phenomenon in each of the cases. Moreover, the data was quantitatively analyzed according to the developed method described above. The framework categories and examples of excerpt of each case, group of managers, organizational level are structural gathered in Tables 5, 6, 7, 8, 9 and 10 and are referred to in the text to prove different phenomenon in the organization. The use of the group of managers instead of a single title for every excerpt gives an improved overview of the management opinions at different organizational levels. Moreover it ensures keeping the anonymity of the hospital and their employees. The excerpts about the national level were fewer and were therefore excluded from the table. However, DC at a national level mostly referred to the national organizations of ICU and infection physicians, who made large efforts to gather important medical information and treatment of the COVID-19 patients and to spread the knowledge to other physicians through webinars once a week as the chief medical officer at case A expressed:

“The Swedish Association of Infectious Disease Physicians has taken on a great deal of responsibility and has held regular webinars with knowledge updates with leading researchers and clinicians in this field.” (Chief medical officer, case A).

Some DC was about the prognoses from the National Board of Social Affairs and Health and the public health authority that was helpful especially towards the end of the pandemic for example:

“During the late spring (2020) and just before the summer, more scenarios are brought up that were sort of adapted based on different regions that you could then work with” (The chief of staff at regional level, case B).

Thus, the SC excerpts describe lack of information and the continuously changing information from Swedish authorities for example:

“Quite shaky at first. Slightly different message. Message not coming … We felt it was messy”. (The chief of staff at regional level, case B).

**Fig. 2:**
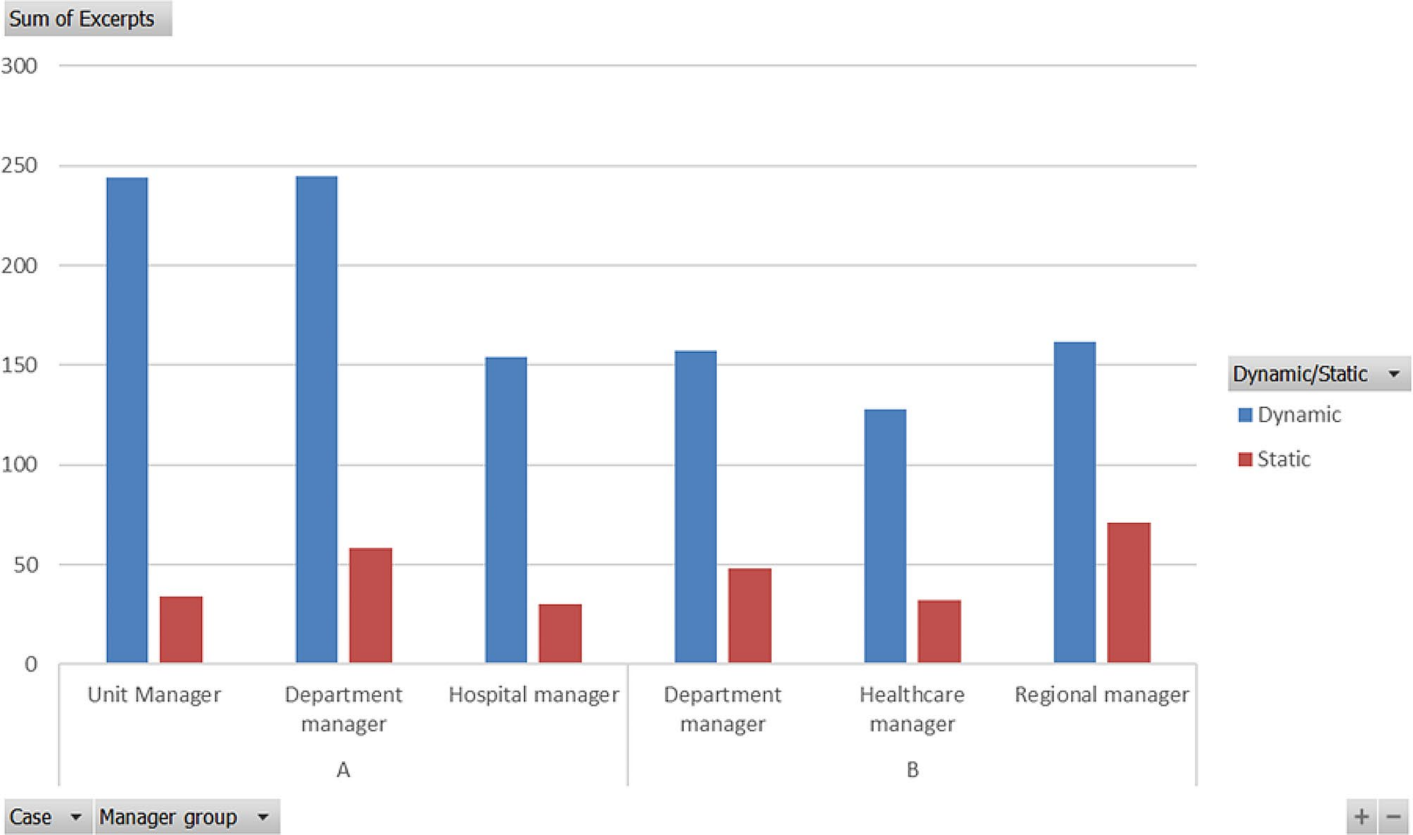
Number of excerpts/group of managers

Figure 2 visualizes the excerpts of each of the units and department managers of case A and there were about double as many as the excerpts of the hospital management. At case B the healthcare management had a slightly smaller number of excerpts. This needs to be remembered during the semi-quantitative analysis. Moreover, Fig. 2 envisions that the managers find the organizations to be more dynamic than static. The hospital manager and unit managers in case A and the healthcare managers in case B have proportionally fewer SC excerpts.

**Table 5:** Case A - unit managers

	Framework categories	No. of excerpts	Example of excerpts across framework categories
DepLev	Sens	52	During wave two [of the pandemic] another patient clientele came to us. Then the elderly was very sick.
	Seiz	54	We introduced morning, lunch, and afternoon meetings, because there was also a need from the staff - and we introduced that too - where we had the opportunity to meet and get information out.
	Trans	70	So now we have some places in ward 23 for high flow.
	NonSens	5	I believe, we patted ourselves on the back, thinking we can take this and it’s so simple and then something happened that we hadn’t imagined - so everyone was a bit shocked at the beginning - what happened here?
	NonSeiz	5	There were some pressured groups were already there - intensive care unit staff and surgery staff didn’t really get along - and there were a lot of old schisms.
	NonTrans a	11	No one really got an introduction, but you had to learn that kind of work shift.
	NonTransb		steal stuff
HospLev	Sens	10	So, we still had to bear in mind that it could have been much worse.
	Seiz	29	We are quite close in our [management] lines up [in the hierarchy], it’s me and then I have my business manager, then there’s the CEO. Like the CEO, you know - it’s not anonymous when you are in a small hospital [as in this case].
	Trans	21	The supply then has – after all, they come up every day and refill.
	NonSens	1	uncertain about materials and all – but it’s a different story – a new disease – no one knew anything about anything.
	NonSeiz	7	…had difficulty with care places at the hospital and many patients remained in the emergency department. The situation we entered the pandemic with was worrying.
	NonTrans	3	But the second wave we got no external resources at all.
RegLev	Sens	0	
	Seiz	2	It has been quite fast to get those decisions through.
	Trans	6	Covid ambulance redirection it was yes.
	NonSens	0	
	NonSeiz	2	In wave two [of the pandemic], politics wanted to take over and try to get some of this back then and like controlling everything - then suddenly it became much slower - it was slower to get care places in wave two than during wave one.
	NonTrans	0	

**Table 6:** Case A - Department managers

	Themes	No. of excerpts	Example of excerpts across themes
DepLev	Sens a	42	It was quite - it was really this sense of uncertainty (it) was enormous - a lot of anxiety in the air - especially for the doctors. So, this was you know with protective equipment and that some doctors didn’t even want to go to the patient - it was very, very chaotic.
	Sens b		This is not a crisis this is a pandemic. It is not a crisis event. It’s not a point it’s a line.
	Seiz a	58	Acting in the boss’s spirit, but it gives them freedom and their own power in carrying out their job.
	Seiz b		Yes, it has gone extremely quickly in acute somatics because we have single rooms there, so we never end up in those situations. We talk all the time in cohorts and then each single room is a cohort, which means you can change from one minute to another.
	Trans a	50	But, on the other hand, we have said that you call out for help if you forgot something when you go into the room and investigate so that you don’t bring too much in because everything must be washed - so it has definitely changed a lot of things.
	Trans b		Here we went from 160 to 320 people [staff] in three weeks and you can just imagine the group dynamics—and a four 100% increase in intensive care unit production here—and the intensive care unit that the hospital had back then when this started.
	NonSens	0	
	Nonseiz	1	It wasn’t clear to us what operational targets we have and what we must work towards, so I’ve had quite a long journey just to understand who is responsible for targets. Well, it is actually the managers who are responsible for the targets and then they must try to get all their employees to understand these targets. So obviously it’s challenging for us.
	NonTrans	0	
HospLev	Sens	18	And the unpleasant thing is that when I talk about this, I get goosebumps all the way down to my knees. I remember so well when this came out and we started discussing it and we slowly started—it probably took a day or two for me to really take it in.
	Seiza	44	With a brave manager at the hospital, and a bit of luck with timing with the right person in the right place, we were able to get momentum going and get going.
	Seizb		So actually, I would like to say that in terms of places and such we have somehow taken a position almost daily on the situation of how we can balance the flow in the best way and take care of the patients. And then we worked with our strategic meetings where we made decisions. There have been decision forums every day when we have been in reinforcement mode and since then, our management team has been in hospital crise mode three times a week.
	Trans	21	Hospital management meeting we have on Teams, but I think there should be both opportunities. It helps - it does. It is not at all so stupid to be able to have digital [meetings].
	NonSens	1	It should have been over before the summer - oh dear - how hard could it be? Who knew. Oh. Uh-oh. So many times, I’ve been wrong in what I’ve believed about this – yes, we’ve learned here that we can’t predict the future - it always turns out in some new way.
	NonSeiz a	21	But I must have tools that keep the staff on the line and continue to perform. We don’t have such tools in healthcare - because we have never rigged for it.
	NonSeiz b		And then there has been a lot - a lot like contractual issues around how to do - how to move staff - you must change schedules and sort of dialogue with the unions around this and there have been compensation issues linked to this because it hasn’t - that has not been so popular to switch - switch and help in other areas of activity.
	NonTrans	5	Perhaps I thought that they were almost overstaffed - there were, as it were, too many people, especially in anaesthesia, my opinion is that it turned out that way.
RegLev	Sens	2	No, that came from statistics - there were statistics from the region - to all hospitals - to intensive care units.
	Seiz	7	In the third wave they have agreed more on how we should scale up and balance the care between our different emergency hospitals - so that we help each other and get a reasonable load on all hospitals as well - so it has become that we have gone in rhythm with the other hospitals and when it has been necessary, we have also had to step up.
	Trans	2	And it would never have worked if we had tried to bring it along or in some other way - but that was where operational managers and intensive care managers collaborated almost on a daily level - several times a week. And this potential that exists in Swedish healthcare and in the region, not least with cooperation between the hospitals instead of them competing as if it were a market.
	NonSens a	4	Reality and the hospitals solved the problem - and they came later and said that you have done this, do that. And it was like - they came again and again and looked at how it turned out and blessed it afterwards.
	NonSens b		Here you have a curve that just rises and rises and rises and it becomes a completely different dynamic and this continued for weeks, months, six months - and the whole healthcare system will be involved. Everywhere, right up to the health centres and the nursing homes and the emergency departments of the emergency hospitals and the intensive care, and it was like a total heart attack of information.
	NonSeiz a	19	And the incentive for why it has not been done and has not been given to us by the staff who have worked as nurse anaesthetist and anaesthetist in the private sector who have rolled on and run ASA1 and ASA2 hips and knees in parallel to keep their production.
	NonSeiz b		In wave two and three, different degrees of political dynamics and divide and rule entered the whole thing, and we have had varying results and have managed significantly fewer patients.
	NonTrans	4	…which is not so well we may have to wait and see what the region says and such - no answers came from the region - they are busy thinking about a lot of other things - or just drowned in questions and meetings.

**Table 7:** Case A - Hospital managers

	Framework categories	No. of excerpts	Example of excerpts across framework categories
DepLev	Sens	2	So, it was a lot about going back to the facts – getting help from experts.
	Seiz	1	They just had it completely clear - suggestions on how to respond and they know their business - like running water and on their five fingers in the smallest detail, and then the internal medicine they follow then.
	Trans	6	Change the way they receive patients several times.
	NonSens	0	
	NonSeiz	2	Many who come from other cultures who actually also have a little difficulty with the language and where we have had a dominance of those groups, there it has been the most difficult. They have been the most afraid.
	NonTrans	0	
HospLev	Sens	30	You can say when the first wave rolled in, it was a bit of a shock experience, I think it was an acute crisis in some way that then became protracted and it left a lot to be desired, you could say, and then…
	Seiz	45	Well, you could say that for this type of crisis, we probably weren’t prepared, but we had enough tools to start it all up, then we’ve developed agile in this, you can still say we started.
	Trans a	33	So that we were very early in setting up for it and with the fact that all elective care was basically shut down, then we freed up resources for paramedics, counsellors, psychologists, physiotherapists for that matter as well. And some went and helped and looked after the patients and some looked after the staff so to speak – so that’s how it is. That’s how it still is.
	Trans b		We rebuilt the hospital in large parts. We cancelled elective care, and we scaled up the care places [in wards].
	Trans c		But it is probably also the case that a lot of other things are set aside. So that you can - you dedicate - you put a lot of focus on this.
	NonSens	0	
	NonSeiz	6	It is very heavy for other care units; we have a very small intensive care capacity.
	NonTrans	2	It’s very interesting and see, like the first wave, how we sit close together, no one is wearing protective equipment and work like that. With the pandemic.
RegLev	Sens	4	There you estimate different scenarios, so to speak, and decide that, ok, what kind of capacity are we going to achieve.
	Seiz	23	In that way, but now we sort of make decisions about our volumes, how we sort of coordinate ourselves, ambulance redirections, we work a lot with load balancing. As a logistician, this is how we manage flows in the region. We’ve never worked like this before, so it’s completely unique from a regional perspective.
	Trans	4	Since the second wave and the third wave, now there we don’t get any directives from RCC, so that’s what happens, there is no ordering from that direction, but all decisions are made in the production coordination group.
	NonSens	0	
	NonSeiz a	16	We had introduced a new model without actually having introduced it properly, but it was introduced in connection with… and then in the second wave there was a discussion about whether we can really work in this, which is intended for major accidents and chemical accidents and as well as special events for a short time.
	NonSeiz b		After all, they have the ultimate responsibility for their hospitals, and I think they realized they didn’t really have the opportunity to influence that they needed to be able to lead the business. And that it was sometimes too slow.
	NonTrans	2	In fact, this is how it would have been a meeting - there is a forum with an abbreviation called… The disaster and preparedness committee or something like that would have been yesterday. But it is postponed because we are in the middle of the third wave. So that if it had been, I would have been able to answer the question. But I think they will update this plan.

**Table 8:** Case B - Department managers

	Framework categories	No. of excerpts	Example of excerpts across framework categories
DepLev	Sens	8	We who are in the front line, it was very tangible for us.
	Seiz	17	We’re pretty good at ad hoc solutions too. It is one of healthcare’s stronger areas of competence, naturally the origin of the fact that we have a certain unpredictability in the organization and for every single patient.
	Trans	30	But he got his… the covid wall that is talked about, he had to build up and such. And since then, we have rebuilt a lot more, it was only the first wall that was built, then even more has been built to deal with the infection.
	NonSens	2	And we … this fear around the disease has subsided in a different way, because we know more. It was something that took on quite large proportions, and rightly so, initially, because we knew so little about it.
	NonSeiz	3	But what we saw from the reassigned staff that we got was that it’s not… it’s not easy for an employee who works in a nursing department to start working in an emergency department. There were a lot of people who almost turned away at the door, like “By God, I can’t work here, it’s too messy and it’s too ad hoc”.
	NonTrans	8	…not so functional to open a completely new care unit from scratch.
HospLev	Sens	6	A target image exists. That target picture can change. And it has been very clear during this part since local crisis management came in.
	Seiz	39	But it was early. It was before there was local special healthcare management, because before that there was something called a coordination group or something like that.
	Trans	22	We have almost only digital meetings, and we have limited the number of seats in the staff room and people keep to themselves. Yes.
	NonSens	4	Perhaps it is also difficult to get a little attention from the organizations where the pandemic had not yet reached.
	NonSeiz	3	But we were a group with a small mandate, so we only had mandate over our own, which made it difficult to navigate.
	NonTrans	2	And that created a bit of internal conflict, because our staff came and transported low-risk patients with high-risk protective equipment, which was of course… pedagogical, it became crazy.
RegHealthLev	Sens	0	
	Seiz	5	The health care regional management information, as it is now called, and it is every other week for the entire county’s business managers. It is something that has developed, because from the beginning there were no meetings like that, and we didn’t have any before the pandemic. There we probably had too many … for the hospitals and something like that.
	Trans	2	Yes, then there was a decision to redeploy personnel.
	NonSens	0	
	NonSeiz a	15	Who manages the hospital?
	NonSeiz b		We worked like some kind of guerilla warfare… It was incredibly unpleasant. It was very boring. We got a lot of criticism for it from above and were told not to speak loudly and everything. Terrible. Yes, it was.
	NonTrans	1	So that an infection 2 [unit] was set up there. It was used, as I said, not fully… certainly not fully.
RegLev	Sens	5	I would say that this research group at the university was healthcare hygiene. And it is not because it would be the organization care hygiene, but it is because care hygiene has a care hygiene senior doctor who is partly a driven researcher and partly has contacts with this research group. It was like personal contacts in combinations with…
	Seiz	6	And there is also a pandemic plan, but it is clear that they have learned so much that the new plans will be much, much better of course.
	Trans	7	…in the same vein, I pressed that we need to have more emergency departments. So then there were premises adjacent to the emergency department that have been planned for quite some time to expand. So, I was released that money, 8.4 million [SEK], to complete an additional clinical part to conduct emergency medical care.
	NonSens	0	
	NonSeiz	10	Yes, there was an emergency preparedness plan. No, it was not used. And we have long pointed out that it does not make sense against a pandemic. We have pandemics. Not really pandemics, but we have the flu every year. We have long pointed out how poorly prepared we are for the flu.
	NonTrans	0	

**Table 9:** Case B - Regional health care managers

	Framework categories	No. of excerpts	Example of excerpts across framework categories
DepLev	Sens	0	
	Seiz	1	So, it has gradually emerged empirically.
	Trans	5	Established a neonatal intensive care unit at home as well. Thus, even premature children, if they feel safe, should be sent home with parents, with support so that they can be sent home much earlier.
	NonSens	0	
	NonSeiz	0	
	NonTrans	0	
HospLev	Sens	9	Because there has periodically been significantly less pressure [incoming patients] in the emergency department, and we have figures for that.
	Seiz	14	I’m thinking of health care hygiene, which was very good at informing at department head meetings, on the intranet, showing … Because it was changing all the time with mouth guards and FFP3 and everything they were called, and what’s current. So information and information and information.
	Trans	26	When we opened to twelve places from… or on the intensive care unit 2, so it was a decision that I made in the management together with the intensive care, because we saw that there was danger on the way.
	NonSens	0	
	NonSeiz	1	Without the same principle… That is to say that the healthcare must, regardless of whether it is war, reinforcement mode then, carry out the same activities with the same management… And then it became very difficult at times… We are line managers as well, so is this a regional staff issue or is it regular business?
	NonTrans	1	Yes, before the preparation for the second and third wave, it was then established that it does not work so well…
RegHealthLev	Sens	7	Oh, my God, 130 treated in the hospital at the same time, of which 50 in the intensive care unit. I think that’s what we said. And then we said that we must do this. So, we were mentally prepared for it, but concretely.
	Seiz a	18	To deal with a pandemic, that is to run healthcare. It is not this kind of crisis management organization at all. But so, it was us then in what is… Yes, that is, my management group, that is, we who are responsible for healthcare. So, we were the ones who decided that we must… This is what we must do.
	Seiz b		And very quickly we started having information meetings … Once a week we have had operational safety meetings with information then from infection control and health care hygiene where they have given a lot of information about everything.
	Trans	12	So, they didn’t build it right away, but the department existed. It was empty. It would be furnished, and all the materials and all that.
	NonSens	2	“It might not even come here.” That was the attitude they had.
	NonSeiz	5	During the pandemic, we had the first turn, from about week ten [of the year] onwards, a healthcare management with also the director of healthcare as management. And then all of us area managers were involved in something that was probably not actually in the plans, but it came so quickly that we had to act in a different way because we had no current plans for how we were going to… So, the whole healthcare ended up in a separate group of staff.
	NonTrans	7	The messiest thing about it was the staffing of it, because we had to get staff from other parts to be able to staff these infection units. It was a bit messy, I can say.
RegLev	Sens	6	We have received the forecast. We are still receiving forecasts for various incidents depending on the spread of infection, the state of infection. And so we know that [inaudible] from the moment of infection until you get sick and a certain proportion then needs hospital care and a certain [proportion] needs intensive care. So there are pretty good models to get a rough idea of ​​what it will look like a few weeks ahead even.
	Seiz	13	And I feel that it works, because we fix it without arguing about what the crisis management organization should look like, so we fix care in any case. A bit like that.
	Trans	14	But it is clear that very quickly a lot of care hygiene went into it and had a very important role in everything from testing and use of protective equipment and so on. We had excellent cooperation with infection control and care hygiene.
	NonSens	1	So, during the spring when everything started, we knew very little about everything. The disease, where it would go and so on, and so on.
	NonSeiz a	11	But these regular disaster and crisis management organizations, they are not adapted to deal with pandemics.
	NonSeiz b		I think that then it would look the same as usual, and that is the principle of similarity we should use, I think. That if we manage healthcare in this way and manage, and this management structure we have. Then it is the one that should be there. But it may need to be reinforced.
	NonSeiz c		It was probably because we didn’t have an up-to-date disaster or pandemic plan. Then we also changed the organization in 2019. Transitioned from an old management model to a new one, and then we didn’t have time … We hadn’t had time to find a new plan for it.
	NonTrans	3	And certainly, there has been frustration towards us management that we have not made clear enough decisions and we have not communicated them, and so on. So there has been some irritation and frustration as well. It has been.

**Table 10:** Case B - Regional managers

	Framework categories	No. of excerpts	Example of excerpts across framework categories
DepLev	Sens	0	
	Seiz	4	If you activate the right people, there are very, very good opportunities to increase resources, for example, in intensive care. I mean, all the colleagues we have there, it is their whole profession to be able to adapt capacity according to the requirements.
	Trans	9	And on the spot spread out those who know intensive care, and bring in a little more people, but that you still had to keep your place, so to speak.
	NonSens	0	
	NonSeiz	0	
	NonTrans	0	
HospLev	Sens	0	
	Seiz	7	And those assessments are made by local special healthcare management at each hospital. So, when you see that it is at a steady level with covid patients, and maybe starting to reduce it. Yes, but then you can scale up a bit. So.
	Trans	3	In the first wave, a couple of doctors from the intensive care unit are sent down to [another hospital] to study these patients, to try to understand: “What do you actually do with them?”
	NonSens	0	
	NonSeiz	2	And then every business manager is left on their own.
	NonTrans	2	Excess capacity as far as intensive care places were concerned.
RegHealthLev	Sens	0	
	Seiz	4	So, I talked about working under uncertainty and how to deal with it. So, we put a lot of energy into what you said as well, that “Ok, now it’s like this. There is a lot that is unknown here, but we still must function. But we must be prepared to change. Today this applies. But these are uncertain facts. And everyone must be prepared for them to change by tomorrow.”
	Trans	4	Well, my employees have been there (at the hospital).
	NonSens	0	
	NonSeiz a	11	At that time, we had not established local crisis management in [name of city], because… well, for various reasons, there was not such a… lack of knowledge in this matter of what is called crisis management, or special healthcare management, that you have not really understood the point of it.
	NonSeiz b		And that then led to confusion, difficulties in the flow of information. Local level didn’t really understand where the decisions are made. So, it became very unclear. And it’s about the fact that far too many people didn’t understand how this was supposed to work.
	NonTrans	4	…but I still can’t interpret it as anything other than an active decision.
RegLev	Sens	35	So, when I say that we create a common situational picture, then we try to capture as many aspects as possible, in terms of staff loss, patient inflow, occupancy rate. So, what you need to capture, but also [need to] forecasts from infection control, forecasts from healthcare hygiene, forecasts from logistics.
	Seiz	48	The area of ​​crisis preparedness is very much regulated by law, which is the task of various authorities to prepare for crises. And it is based on risk and vulnerability analysis and will land in a crisis and disaster medicine plan that we will have with a crisis management organization, with different roles that are determined that will be trained and practiced. That is the basic idea.
	Trans	29	I understood that they could set up lab analysis capabilities. So, we requested that from them back in March, or something like that, I think. Yes. And that meant that they… And they’re a bit quick-footed too, here locally. It didn’t take a lot… weren’t forced by a lot of agreements, but they started and planned their… And then we were able to direct it to be used out in the municipalities and in primary care to a much greater extent, I think, than anyone elsewhere.
	NonSens	5	There was very little information that went to regional special healthcare management.
	NonSeiz	22	If you talk from a crisis and disaster medicine perspective, it is not ok that such a large actor as a region does not have the ability to ensure that you follow your plans and that you have trained and practiced personnel. So that was a big shortcoming that we saw early on.
	NonTrans	9	The concern is that when you join a crisis management organization, you take off your old hat and put on a new hat and step into a different role. And that role is clearly described how it should act. And if everyone had done that, then I think there would have been greater clarity in the entire organization.

**Fig. 3:**
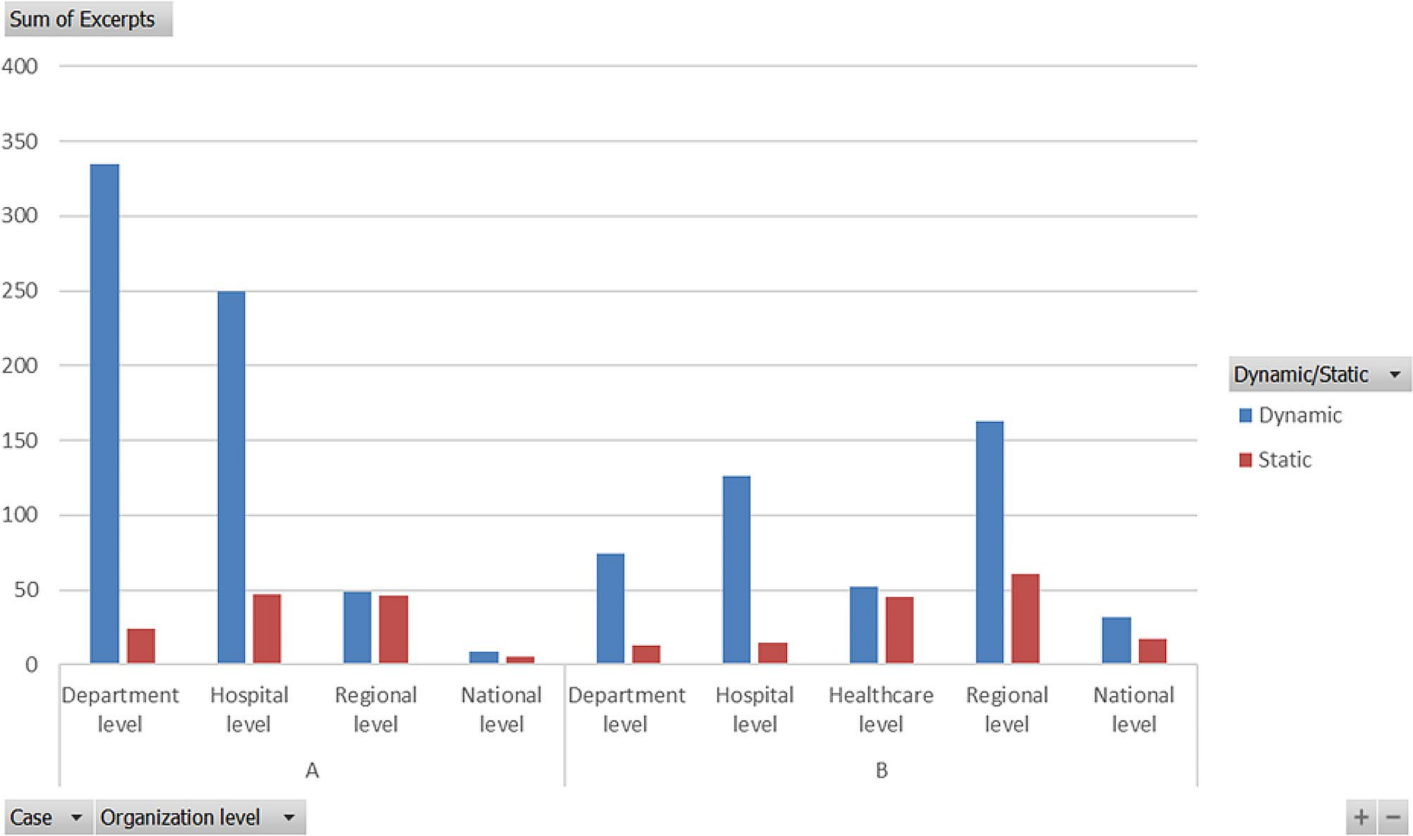
Number of excerpts/organization level

Figure 3 visualizes the number of excerpts per organization level to show their dynamically respectively statically behavior during the pandemic. The department level in case A received the highest number of dynamic excerpts followed by the hospital level, however, the hospital level has a higher proportion of SC. The highest proportion of static behavior, showing nearly the same number of excerpts as DC, are found at the regional level. The examples of criticism was that they lately understood the severeness of the COVID-19 pandemic (Table 6, RegLev: NonSensa), pushed to work use the NATO standard even if it was not implemented (Table 7, RegLev: NonSeiza) and kept the structure of crisis management even when the disasterwere prolonged. However, further into the COVID-19 pandemic RCC lost power towards the normal group of hospitals directors, which made the hospital managers more positive towards the regional level. (Table 7, RegLev: Trans). Examples of criticism from the department managers towards the regional level appears later in the COVID-19 pandemic when the politicians changed focus and made the cooperation over the region work less effective (Table 6, RegLev: NonSeizb). The politicians also caused dissatisfaction among the professionals by building an ICU at a fair hall outside the hospitals which was never used.

In case B the highest numbers of dynamic excerpts were at the hospital level and at the regional level, but the proportion of SC at the regional level was higher. The healthcare level had the highest proportion of SC with slightly the same number as the DC correlative to the situation at the regional level at case A. The healthcare level got criticised both from the department and the regional managers, for example one regional manager’s questioned the active decision at the healthcare level to have their own regional crisis management beside the RCC and that they did not start an LCC at the case hospital (Table 10, RegHealthLev: NonSeiza; Table 8, RegHealthLev: NonSeiza). Further, the regional management had a high proportion of SC especially from the healthcare level, because of the regional levels strong statement of a contingency plan that maybe was not appropriate in a pandemic (Table 9, RegLev: NonSeiza). The regional healthcare level pushed for management more as usual as in line with the hospital managers at case A (Table 9, RegHealthLev: Seiza). Thus, the department managers started an local manager group at the hospital for practical decisions and needs without any mandate and official agreement (Table 8, RegHealthLev: NonSeizb).

**Fig. 4:**
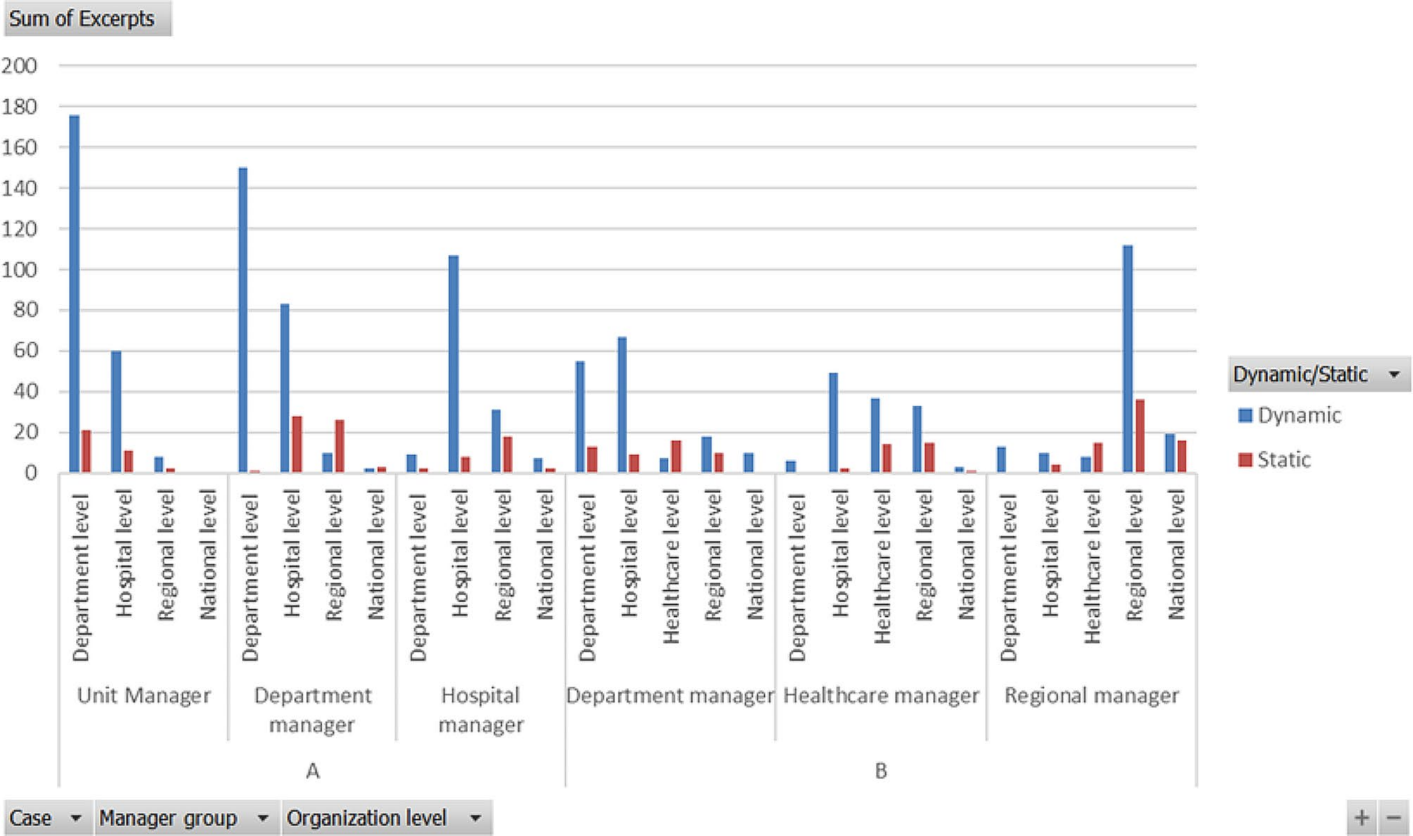
Number of interviewees excerpts for each group of managers/organizational level

When looking closer of how different management groups assesses each organization level (Fig. 4) the cases differ even more. In Case A the managers consider their level with positive eyes as well as the level nearest above, for example when the department manager group praised the hospital manager for his braveness (Table 6, HospLev: Seiza) or when the department manager group talked about their thoughts of getting the employees to act with the managers spirit (Table 6, DepLev: Seiza). However, the most SC also appeared for the level directly above, for example, that the department level underestimated the COVID-19 changings (Table 5, DepLev: NonSens) and the lack of tools for keeping employees at the working place in a stressing environment (Table 6, HospLev: NonSeiza). However, the unit managers evaluates the second nearest hospital level dynamic and comment on the short distance to the hospital director, known by everyone (Table 5, HospLev: Seiz).

All manager groupsof Case B seem to be self-critical and considered their own level as being somewhat static, for example the department managers reflection that the idea to start a new department was not the best choice (Table 8, DepLev: NonTrans) or the regional managers reflection of their poor management when the healthcare LCC was not started in the beginning of the pandemic (Table 10, RegLev: NonSeiz). The regional manager group seem to be self-confident about their own level (Table 10, RegLev: Seiz), but the number of excerpts from the regional managers reflected that the healthcare level has higher proportion of SC than DC caused by the special crisis management group at healthcare regional level as described before. The healthcare manager group have a high number of dynamic excerpts towards the hospital level, who they found transformed by building additional beds at ICU (Table 9, HospLev: Trans), but do not have many comments about the department level. The proportion of SC is high from the healthcare managers towards the regional level arguing that a pandemic need to be managed by normal healthcare management (Table 9, RegLev: Nonseizb). Caused by interviewing the regional management of case B, the excerpts about the national level are present in higher numbers – both positive and negative.

**Fig. 5:**
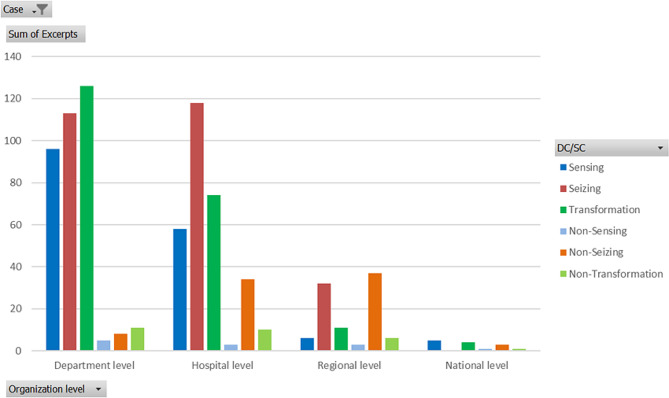
Excerpt/organization level, case A

At hospital A the sensing and transformation occurred more frequently at lower organization levels (Fig. 5) with a descending occurrence at higher levels. At the department level in case A they listened to the international network and because of their closeness to the production they saw the changing number of patients and clearly sensed the level of worry and stress on the organization (Table 6, DepLev: Sensa). Further, they early on realized that a long duration pandemic made the situation different from other disasters (Table 6, DepLev: Sensb). The proportion of transformation was high and for example they managed an increase in employment at the ICU from 160 to 320 (Table 6, DepLev: Transb). Moreover, they changed working procedures, for example agreeing on an allowance to shout out into the corridor when you needed something to avoid taking the Personal Protection Equipment (PPE) off and on again (Table 6, DepLev: Transa). Examples of non-transforming capabilities were overusing PPE, the infection spread between employees, the shortage of employees at the critical units and the shortage of training before work a shift at a new position (Table 5, DepLev: NonTrans; HospLev: NonTrans).

At the hospital level they sensed the employees’ anxiety and worries about the risk of infection for themselves and relatives and the knowledge shortage when moving to other tasks and transformed by arranging psychological help for the employees (Table 7, HospLev: Transa). Moreover, they helped with recruitment, moved employees to the units needed, built education and hygiene rounds, and started and stopped planned surgery several times (Table 7, HospLev: Transb). The meetings became digital and the number of employees in the coffee rooms at once was reduced and they reconstructed several departments. The non-transformation was rather high at the hospital level, possibly a sign that transformation was too late or not large enough (Table 7, HospLev: NonTrans).

The seizing was found equally at department and hospital level. Hospital, department, and unit levels of case A increased the frequency of meetings to daily or even more. (Table 5, DepLev: Seiz). The unit managers used the existing dynamic quality of the organization including single rooms at the wards (Table 6, DepLev: Seizb), the united management of all wards and the knowledgeable management of ICU to take necessary decisions and execute them. The cooperation between the hospital departments increased and there was a focus on healthcare and all other questions were not prioritized (Table 7, HospLev: Transc). The non-seizing was the most occurring static behaviour, and it increased in occurrence with higher organization level. Thus, the structure for moving employees to even out the pressure, for example agreements of compensation and individual education, were not in place and were not working properly (Table 6, HospLev: NonSeizb). Moreover, department managers responsible for the reduced planned healthcare were not allowed to use their free time to develop their organization and they also commented that the focus of staffing at ICU was too high and that the decisions about the start of surgery in between the waves came to late (Table 6, HospLev: NonTrans). The non-seizing towards the regional level was higher than the seizing. The criticism was that there was too little capacity at ICU in the region, neither agreements for cooperation between the public hospitals nor between the private and public hospitals were in place (Table 6, RegLev: NonSeiza). Moreover, the contingency plans structured methods of communication, built for short term disasters, caused a lot of questions, especially during the first wave and no one listened to the managers respond about what happened at the hospital (Table 7, RegLev: NonSeizb). The excerpts of seizing described the appreciation when the production group later became more powerful. Moreover, the ICU managers met over the region at a regional level and made decisions (Table 6, RegLev: Trans).

**Fig. 6:**
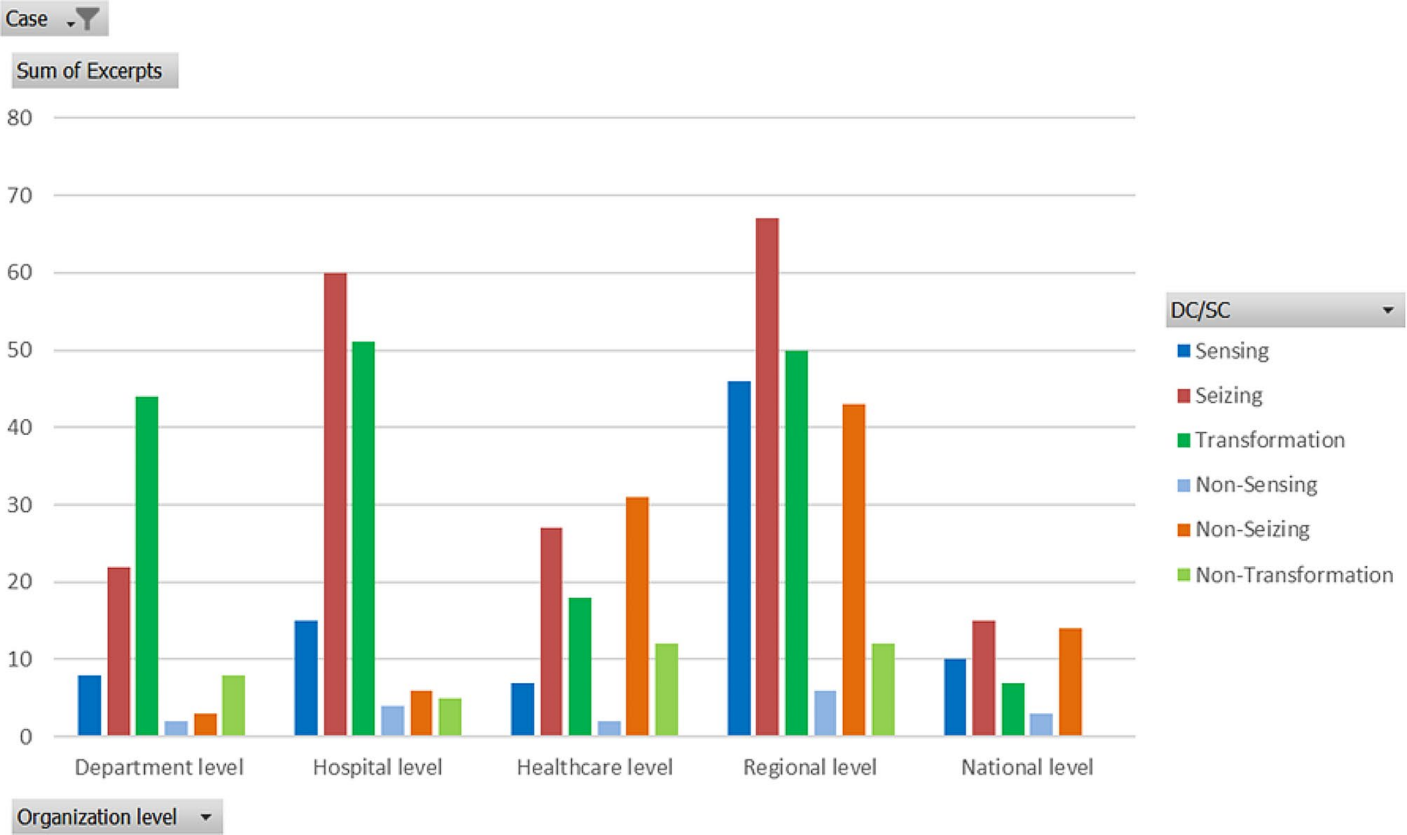
Excerpts/organization level Case B

The DC and SC excerpts pattern/organization level in case B were different compared to case A (Fig. 6). Instead of the highest number of excerpts about transformation near the production, the transformation in case B seems to have been high at department, hospital, and regional level and lower at the healthcare level. The number of excepts about sensing was surprisingly highest at the regional level, which possibly is due to the highly experienced and knowledgeable regional chief hygienist physician’s high and his trust and a good international network (Table 8, RegLev: Sens). Moreover, the chief hygiene physician contributed to merely transforming the organization by decisions to decrease the infection between employees and at elderly homes (Table 10, RegLev: Trans), which also caused the high number of transformation excerpts at the regional level. The number of excerpts for non-seizing is high both at the healthcare level and the regional level due to the earlier mentioned argumentation unclearness of documentation about where decisions were made (Table 10, RegHealthLev: NonSeizb) and this meant a focus of seizing at hospital level. The non-transformation was rather high both at the healthcare level and at the regional level.

## Discussion

The discussion is divided into discussion about the developed research method and discussion about the result of the multiple qualitative case study.

### Research method

The research presented developed the use of DC in a qualitative deductive analysis of interviews and is novel especially in healthcare organizations. The data were analyzed with framework categories (microfoundations)of DC i.e., sensing, seizing and transformation following other scholars’ approach, e.g., Teece et al. [18]. , . In addition to this proven application of DC the interviewees’ excerpts, which narrate a static behavior, were coded as framework categories (microfoundations) of SC i.e., non-sensing, non-seizing, and non-transformation to increase the visibility of malfunctions in the disaster management analysis [22, 23]. The coding of both DC and SC contributes to a more encompassing analysis of the organizations’ development during the COVID-19 pandemic. The introduction of SC shows important insight also into occurrences that may reverse the movement towards transformation of the organization. Further, the coding was divided by management group and organization levels, which revealed a visualization of the dynamics in between the management levels which had not been found in earlier research. The excerpts were only coded once and therefore the qualitative analysis could partly be quantitative even if some excerpts might contain several items. The method was used to analyze multiple cases and successfully revealed differences between the organizations when using this developed technique of analysis.

### Multiple case study

The professionals working in production in both cases clearly sensed the situation when the COVID-19 patients arrived and the organization rapidly transformed to save lives, in line with research by Teece [34] and Ohrling et al. [8]. The early sensing at department level in case A, due to an international network made the organization transform even before the first patient arrived. These occurrences of sensing made the response to changes in demand possible even with high focus on operational tasks, despite such situations can be proved to be non-resilient [12, 34].

All DCs were, according to the managers narratives, present at the department level in case A and the occurrence of high seizing impeded the suboptimizing of the local unit over the whole organization that could occur with strong sensing and transformation [18, 29]. Moreover, seizing, which Furnival et al. [12] mean is advantageous when working with continuous development, might be a sign of a higher need of continuous development in a long-lasting pandemic than in a short crisis.

The highest occurrence of sense in case A was found at the department level and led to high expectations of seizing and transformation, which according to our research was not delivered from the regional or national level, which is aligned with situations described by other scholars [8, 18, 29]. In fact, the quota between the number of interviewees’ excerpts/framework categories of seizing and non-seizing decreases with higher organization level in case A, which suggests that the top management was less dynamic. The low sense at the regional level made the mistrust high, possibly because that they appeared arrogant and unwilling to change, in line with the study of Furnival et al. [12]. When the RCC was overtaken by the hospital managers, this production group made the organization more dynamic, and the sense of the situation was more easily transferred to the regional level. Later, when the politicians started to interfere with the organizations, the cooperation between the hospitals decreased, which resulted in suboptimization of the local units in the organization, which decreased the overall organizational efficiency, as also expressed by Ljungquist [29].

However, the situation in case B, where the non-official department management group originated in the absence of a strong hospital manager or a working LCC, became different. The department management group sensed the situation and transformed accordingly, which according to Ljungquist [29] and Teece et al. [19] research could cause suboptimization of the regional cooperation as well as high barriers between the department managers and the regional healthcare management. However, because the sense was high at the regional level the barriers between the department manager’s group and regional management were not seen. The chief hygiene physician at regional level early sensed the situation, by his international network and reacted fast, transformed, and successfully reduced the infection rate also outside the hospital. His placement at the top of the organization, far away from the production, was of a less hindrance due to his and his team members’ high frequency and trustful contacts with the organization’s lower management levels. The lower occurrence of sensing in case B, except for the regional level, is probably the cause of the decreased confidence between the organization levels, which is in line with Furnival et al. [12]. Moreover, Ljungquist [29] and Teece et al. [18] discuss that a higher occurrence of seize could mean higher barriers and mistrust both between local units and the units and top management, which is also recognized in case B. The low sense at healthcare level made the mistrust even higher possibly due to the appearance of arrogancy and unwillingness to change [12].

When the knowledge increased, and the COVID-19 infections changed, the transformation continued in cycles, as Eriksson et al. [20] highlighted in their research, for example when the surgery started stopped and restarted several times in case A. Another cyclic change occurred in case B when the launching of a new infection department failed due to problems with staffing. This proved an important learning point for the next step of transformation when instead an old inpatient unit was transferred, which follows the experimentation work described by Pablo et al. [13].

The information flow during the COVID-19 pandemic was enormous especially in case A with the higher and earlier breakout and the recommendations often changed and made the information channels break down. Using integrated information from different sources from different management levels like Ohrling et al. [8] suggest could probably also in our case reduce the amount of information and reduce misunderstandings.

The contingency plans, which the regional crisis management at both cases insisted on following were designed to manage a short-term crisis and seemed to be built according to a static and hierarchical SuC. However, this and other studies reveal a need for more distributed management in a long-term disasters [4, 7, 8]. Reality often differs from beforehand plans and if the plans are followed too strictly the organization will be static and not able to follow the dynamic changes [32]. The regional level’s insisting on sticking to the contingency plan excluded them from supporting the pandemic. Moreover, in case B the contingency plan caused a lot of argumentations about the plan instead of looking at the reality and developing a sound cooperation between the levels in the extended work caused by the pandemic. However, the regional healthcare level in case B insisted on keeping normal management routines, but because of the low sensing at regional healthcare level in case B this did not function. Whereas in Case A this approach worked well. The focus on following the plan in Case B possibly made the management levels less sensitive to the situation [12]. The suggestion from Eisenhardt et al. [11] to have “routines to learn routines” could build a more successful disaster management in a next pandemic.

### Concluding discussion multiple case study

Case A had at department and hospital level well developed and synchronized DCs and managed the high pressure of the COVID-19 pandemic successfully, as foreseen by other scholars [12, 33]. The managers in case A described that they and their employees became more self-confident and took decisions independently, which is in line with the reasoning by Ohrling et al. [8] about decision space as a success factor during the COVID-19 pandemic. The cooperation and trust at department and hospital levels increased during the pandemic, which is in line with research by Ohrling et al. [8] and Pablo et al. [13]. Higher management levels lacked developed DCs, which grew mistrust between the hospitals in the region and the regional management.

However, in case B the seizing and non-seizing were the strongest capabilities, which could be the sign of a concentration and discussion of routines in the overall organization rather than supporting a transforming at department level to save lives. The seen self-criticism in case B could be a sign that the management was malfunctioning, and they were looking for what was wrong at their position. To conclude, Case B coped well with the pandemic, however, they might have had problems succeeding if encountering the higher infection rate, such as in case A.

## Conclusion/relevance/contribution

The method, using a deductive analysis of analyzing with DC and SC, different management groups and organization levels, has successfully been used when explaining the crisis management in healthcare organizations during a long-term disaster as a pandemic. This novel way of analyzing data facilitated a structured and detailed explanation of organizational behavior and has not been found in earlier research.

The case hospitals studied showed major differences, when evaluated with the promising DC-method; In case A the hospital manager was considered by the lower-level managers to be brave and strong and supported the professions sensing, seizing and transformation at the department level. Due to the information developed at profession level, the sensing did not reach the regional disaster management, thus could not appropriately support the transformation and their power was reduced in favor of the normal management and cooperation between hospital managers in the region. However, in case B, where the contingency plans stated LCC were not started, the hospital suffered from lack of management and started their own department manager group to be able to take care of the incoming patients. The seizing was high in the organization with the department management developing their own routines, while the regional level and regional hospital level got stuck in discissions about the best choice of management between disaster management or normal management. However, both cases did use DC’s and the capabilities were synchronized enough to withstand the COVID-19 pandemic at the level needed.

The managerial contributions from thisresearch are in line with other scholars.Crisis management in a pandemic need to be more distributed and dynamic and this view need to be the starting point for top management to develop a contingency plan specialized for pandemics. The pandemic plan should manage to develop routines according to the demand from an ongoing pandemic, develop and use DC’s in the whole organization to support the profession to sense, seize and transform. Moreover, building professional networks could help reaching an early sensing, where two examples are, the one that made case A start early to build capacity and the one at case B that reduced the infection rate, which will give an opportunity to save lives. In a long-lasting pandemic, cyclic and continuous improvement seems to be needed.

### Limitations and future research

A limitation of this paper,, is its potential to generalize the findings from two Swedish hospitals’ case studies to other healthcare facilities or different organizations. THowever, this limitation is somewhat outweighed by the successful intention of obtaining rich data coupled with an in-depth analysis based on interviews with different manager groups’ view of the management at different organizational levels, which contributes an encompassing view of the applicability of the DC framework in health care. Nevertheless, additional research is needed to enhance the promising method’s effectiveness and support its broader development. It is highly recommended to conduct further studies in this area, expanding its application to diverse types of organizations and environments. It would be interesting to supplement the data with further inquiries about the current application of lessons learned during the pandemic. Especially what was learnt about the possibilities for flexible organizations to make multiple transformation to follow the changing environment during av pandemic. Not just that they transformed but also why some managers was able to build trust and avoid power games and negative story telling in the organisation. To summarize it is important that the insights gained from the COVID-19 pandemic should be carefully refined to strengthen disaster management, thus improving our readiness for future pandemics.

## Data Availability

No datasets were generated or analysed during the current study.

## Abbreviations

DC: Dynamic Capabilities
R: Resilience
SC: Static Capabilities
SuC: Surge capacity
DC and SC: Framework categories
Sens: Sensing
Seiz: Seizing
Trans: Transformation
NonSens: Non-Sensing
NonSeiz: Non-Seizing
NonTrans: Non-Transformation
LCC: Local command centre
RCC: Regional command centre
DepLev: Department level
RegHealthLev: Regional healthcare level
HospLev: Hospital level
RegLev: Regional level
ICU: Intensive care unit
PPE: Personal protective equipment
